# Determinants of healthcare expenditures: evidence from Türkiye’s healthcare system

**DOI:** 10.3389/fpubh.2025.1632883

**Published:** 2025-10-09

**Authors:** Hülya Diğer, Kübranur Çebi Karaaslan, Ahmet Köstekçi, Abdullah Göv

**Affiliations:** ^1^Department of Health Management, Faculty of Economics and Administrative Sciences, Erzurum Technical University, Yakutiye, Türkiye; ^2^Department of Econometrics, Faculty of Economics and Administrative Sciences, Erzurum Technical University, Yakutiye, Türkiye; ^3^Department of Public Finance, Faculty of Economics and Administrative Sciences, Fırat University, Elz, Türkiye; ^4^Department of Management Information Systems, Faculty of Economics and Administrative Sciences, İstanbul Arel University, İstanbul, Türkiye

**Keywords:** Türkiye, post-pandemic, out-of-pocket health expenditure, probit, health economics

## Abstract

**Background:**

The lack of a mandatory referral system for service demand in Türkiye’s health system and the belief that unnecessary health expenditures are increasing constitute the basis of this study. This study aims to analyze the determinant role of individuals in health expenditures in Türkiye in two dimensions: household indicators and indicators for the head of the household, taking into account health expenditure policies.

**Methods:**

In this study, the factors affecting households’ out-of-pocket health expenditures are tested with the help of binary probit regression analysis using the micro data set of the Turkish Statistical Institute 2022 Household Budget Survey.

**Results:**

The findings revealed no significant relationship between out-of-pocket health expenditures and variables such as gender, education level, employment status, private insurance ownership, alcohol and tobacco use, ease of access to a health center, receipt of monetary aid, or household size.

**Conclusion:**

According to the results of the research, older individuals spend more on health expenditures. Expenditures increase in parallel with education expenditures and health expenditures decrease as income level increases.

## Introduction

The term “health expenditures” encompasses both personal and collective service expenditures. The former includes costs associated with treatment and rehabilitative care, long-term care, medical goods, and ancillary services. The latter, in turn, comprises public health expenditures and health administration costs, among others. By this classification, health expenditures are divided into two categories: public and private (out-of-pocket, voluntary) expenditures (1). The advancement of technology and the consequent growth in knowledge about health services have contributed to the rise in health expenditures (2). Health policies and the configuration of the health system can also influence health expenditures. Therefore, health expenditures are influenced by cost-containment strategies (3). Changes and transformations in Türkiye’s health system have also played a role in shaping health expenditures.

**Figure 1:**
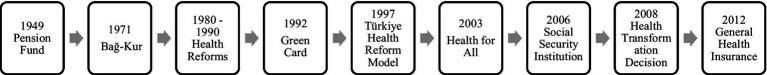
Historical background of Turkey’s health expenditure policies.

The historical background of the Turkish health system has seen the implementation of numerous policies pertaining to the management of expenditure in the context of health services. The report published by the World Health Organization in 2000, “Health Systems: Improving Performance” published by the World Health Organization in 2000, the issue of fairness in financing was included and accessible health for all was emphasized globally (4). This situation has encouraged Türkiye, like every country, to take various initiatives for health financing. On the other hand, it was also stated by the World Health Organization that health literacy is a factor and an outcome in participation in health services (5). These issues and developments have opened the door to some changes in Türkiye’s health financing. In this context, Figure 1 shows the historical background of Türkiye’s health expenditure policies.

A detailed analysis of the health policies designed for the management of health expenditures in Figure 1 reveals that many practices have been implemented to manage the health expenditures of individuals. In this direction, in order to provide financing in health services, the social security activities of civil servants working under Law No. 657 and their dependent families are financed by the Pension Fund established in 1949 (6), while the health expenditures of self-employed groups operating independently (tradesmen, businessmen, farmers and artisans, etc.) are financed by Bağ-Kur established in 1971 (7, 8), and the health expenditures of individuals below a certain income level in line with the minimum wage were financed by the Green Card (7, 8), established in 1992. Between 1980 and 1990, a different approach to financing was adopted and the policies designed by the government included the issue of “*separation of provision and financing of health services*” (9). In 1997, a Health Reform Model was designed in Türkiye, emphasizing “*Social health insurance covering all and improvement in the level of health*” (10), and in 2003, “*Separation of health service delivery and financing*” was underlined among the plans targeted under the title of Health for All (11). Many third organizations (insurance companies) providing financing for health services were gathered under the umbrella of the Social Security Institution established in 2006 (12). In 2008, when the decisions of the Health Transformation Program were announced, a “*General Health Insurance bringing all persons under one roof*” was introduced (13). Implemented in 2012 and still in force today, the General Health Insurance finances the health services of Turkish citizens residing in Türkiye (14).

It is known that policies designed for health expenditures can prevent wasteful spending. In addition to the aforementioned policies, there have been other attempts to reduce expenditures in Türkiye’s health system. In this respect, reducing time and cost wastage in health services is one of the objectives of the “Health Transformation Program” that Türkiye started to implement in its health system in 2003. On the other hand, an “Effective, Stepwise Referral Chain” was highlighted in the “Health Transformation Program” and it was stated that “*We are aware that the majority of the problems faced by patients can be solved in first level primary care and that hospital outpatient clinics are full of such patients*” (11). In this direction, the “Health Transformation Program” aims to prevent unnecessary health expenditures as well as achieve improvements in health services. Based on this objective, the “Family Medicine Model” was put into effect to implement the stepwise system in health services. Following the Family Medicine Model, the referral chain application was launched in four pilot provinces (Denizli, Isparta, Bayburt, Gümüşhane) in 2008. However, the practice was abolished in the pilot provinces and was not put into effect in other provinces due to concerns that it was not successful (15). With the abolition of the referral system, patients assumed a decisive role in expenditures by applying to the hospital of their choice.

There are several studies in the literature on the determining role of individuals in expenditures. These studies support the importance of the referral system in healthcare and state that treating cases that can be addressed in first level primary care at other levels will increase expenditures (16), and that active participation in the referral system (17) and the related process (18) as well as assigning a gatekeeper role to physicians (19) can prevent unnecessary healthcare expenditures. The referral system, which is also called the stepwise system or referral chain, is carried out differently in each country/region. It is managed in line with the health system designed by taking into account the characteristics of the target segment, particularly the welfare level. In this context, the New Zealand Health System requires individuals to comply with the referral system except in case of accidents and emergencies (20). In the Slovenian Health System, family physicians have a gatekeeper role (21). In the German Health System, the service received by patients who do not comply with the referral system is financed out-of-pocket (22).

Unlike other health systems, Türkiye’s health system does not have a mandatory referral system. This context formed the rationale for investigating individuals’ roles in escalating health expenditures and provided the basis for this study. As a matter of fact, in the expenditures of 2022, which is evaluated in this study and viewed as marking the end of the global pandemic (COVID-19), it is thought that individuals played a decisive role as a result of the differentiated health literacy levels during the pandemic. In this context, the letter sent by the Social Security Institution to private hospitals due to overcrowding and missed appointments (23), the study initiated by the Ministry of Health in 15 thousand households to determine the level of health literacy (24), the start of the “Approved Appointment” period in hospitals (25) and the start of the “Routing to Family Physician” practice (26) support these ideas. Therefore, this study aims to identify the determinant role of individuals in health expenditures. Within the framework of this objective, the study was conducted in the case of Türkiye and aims to provide various policy recommendations to health system designers.

There are studies in the literature evaluating health expenditures by focusing on healthcare systems. However, there is no study in the literature that focuses on the determining role of individuals in health expenditures by examining Türkiye’s health system and makes policy suggestions based on the practices in other health systems. This constitutes the originality of the present study. On the other hand, the determining role of individuals in health expenditures in line with the differentiated health literacy levels following the pandemic in Türkiye and the aforementioned practices of the Ministry to minimize this situation make it necessary to investigate the issue. Based on the findings of the study, it is aimed to make various suggestions for reducing expenditures in Türkiye’s health system. The aforementioned issues emphasize the importance of the study and the rationale for conducting it. Accordingly, the study examines the determining role of individuals in health expenditures in the case of the healthcare system in Türkiye.

The main reason for addressing health literacy in health expenditures is the changes and transformations in Türkiye’s health system. Especially in the post-pandemic period, the increasing health demands and the increasing number of health institutions are indicative of some problems. In this sense, according to the results of the study conducted by the Ministry of Health of Türkiye, General Directorate of Health Promotion in 2024, 29.2% of Türkiye has a problematic-limited level of health literacy and 36.7% has an adequate level of health literacy (27). The findings and the research conducted in this sense have formed the basis for the idea that health literacy may have a role in shaping health expenditures. Based on the results obtained from the study, it was aimed to shed light on the determinant role of individuals in health expenditures and the study was carried out. By determining the determinant role of individuals, it is aimed to reduce health expenditures with the recommendations to be made for health policies and the system. This is among the unique aspects of the study.

## Literature review

Studies in the literature on the determinants of health expenditures have been conducted within the framework of socio-demographic and socio-economic factors as well as the health systems and policies of countries. A review of the literature reveals that there are studies examining the issue using different methods. Studies conducted in this direction were tested using regression analysis (28–39), cointegration (35, 40–46), time series (19, 47–49), cross-section (19, 48, 49), least squares method (50, 51), panel data analysis (52, 53), decision tree method (54), Box-Cox transformation model (55), and Log t test (56).

Table 1 provides a general framework for previous studies on the determinants of health expenditures.

**Table 1:** Summary of significant studies on health expenditures and their determinants.

Study	Country (Location)	Method	Data	Result
Gbesemete and Gerdtham (33)	Africa	Regression	Data for 30 African countries	There is a positive relationship between high level urbanization and health care expenditures.
Hitiris and Posnett (49)	OECD	Time series and cross-section	560 observation data for 20 OECD countries for the years 1960–1987	GDP is an important determinant of health expenditures.
Gerdtham et al. (19)	OECD	Time series and cross-section	Data for 22 OECD countries over a 20-year period	Assigning a ‘gatekeeper’ role to physicians in primary health care institutions has been found to reduce health expenditures.
Herwartz and Theilen (48)	OECD	Time series and cross-section	Data for OECD countries for the years 1961–1979	Technology and income elasticity have an impact on health expenditures.
Okunade (55)	Africa	Box-Cox transformation model	Cross-sectional data for 26 African countries after 1995	Population per health personnel, official development assistance and declining income inequality increase health expenditure.
Toor and Butt (35)	Pakistan	Multiple regression, cointegration	Annual budget statement, population and housing census, budget summary and economic surveys on an annual basis	The determinants of health expenditures are GDP per capita, literacy rate, foreign aid, urbanization and crude birth rate.
Akinkugbe and Mohanoe (31)	Lesotho	Linear regression	Ministry of Health, Ministry of Finance and Development, and Bureau of Statistics data for the period 1980–2001	The number of doctors, immunization of children and female literacy were found to be important determinants of health expenditures.
Murthy and Okunade (30)	Africa	Regression	Cross-sectional data from 44 African countries	Non-income factors play a very small role among the determinants of health expenditures.
Wang (29)	USA	Regression	Data for 1999–2003	Four factors were identified as the main factors in the determinants of health expenditures. These are the number of beds, urbanization, the proportion of the population over the age of 65 and the degree of old age within the population.
Cantarero and Lago-Penas (28)	Spanish Regions	Regression	Data for 17 Spanish Regions for the period 1992–2003	There is a correlation between income and public health expenditures.
Herwartz and Theilen (47)	OECD	Time series	Data for 18 OECD countries for the period 1975–2006	Health expenditures become more unstable with aging.
Tang (40)	Malaysia	Co-integration	Data for Malaysia for the period 1967–2007	Income, the price of health services and the proportion of the population over the age of 65 were found to be among the important variables in terms of health expenditures.
Martín et al. (57)	-	-	20 article studies for the period 1998–2007	A review of the literature on the determinants of health expenditures revealed that the common determinants are aging population and proximity to death.
Magazzino and Mele (51)	Italian Regions	Least-squares method	Data for Italian Regions for the period 1980–2009	The number of beds, unemployment rate, real Gross State Product, the percentage of the population with at least secondary school education and the degree of urbanization are found to have a direct impact on real health expenditures.
Prieto and Lago-Peñas (58)	Spain	Regression	Data for Spain for the period 1992–2005	There is a correlation between income and public expenditures.
Samadi and Rad (42)	ECO	Co-integration	World Bank and World Health Organization data for the period 1995–2009	GDP has a long-run relationship with per capita health expenditures. On the other hand, there is a short-run relationship between the number of physicians, urbanization, population under 15 and over 65 and health expenditures.
Boachie et al. (45)	Ghana	Co-integration	Data for Ghana for the period 1970–2008	Urbanization, life expectancy, inflation and birth rate have an impact on public health expenditures. Moreover, real GDP has a positive effect on public health expenditures.
Folahan and Awe (43)	Nigeria	Co-integration	Data for Nigeria for the period 1976–2010	There is a long-run positive relationship between health expenditures and the number of hospitals, physicians and nurses.
Hosoya (53)	OECD	Panel data analysis	Data for 25 OECD countries for the period 1985–2006	Unemployment, time, real GDP per capita, the ratio of female population aged 15–64 to female labor force, and the number of persons per square kilometer have an impact on health expenditures.
Khan and Razali (44)	Malaysia	Co-integration	Data for Malaysia for the period 1981–2014	Out-of-pocket health expenditures have a significant impact on household income and pave the way for the poverty trap.
Olasehinde and Olaniyan (50)	Nigeria	Least-squares method	2010 data from the Harmonized Nigeria Living Standards Survey	Individuals and households in general play a role in reducing health expenditures.
Sagarik (38)	Southeast Asian Countries	Regression	Data for 9 countries for the period 2002–2011	The increasing volume of industrialization and foreign investment have been found to lead to higher health expenditures.
Sinha et al. (32)	India	Regression	Health and Social Security Morbidity Survey data for 986 households in 2004	Out-of-pocket health expenditures are at higher levels among families in the high expenditure bracket. On the other hand, the probability of catastrophic health expenditures is higher among the socioeconomically disadvantaged.
Akca et al. (54)	Türkiye	Decision tree method	2014 data for 35 OECD countries	Life expectancy at birth, number of hospitals, age dependency ratio and GDP per capita are found to be important determinants of health expenditures.
Nghiem and Connelly (56)	OECD	Log t test	Data for OECD countries for the period 1975–2004	Population aging, insurance and technological advancements are among the main drivers of health care costs. However, technological progress has been found to be the main driver of health expenditures.
Tokatlioglu and Tokatlioglu (39)	Türkiye	Regression	Household Budget Survey data for Türkiye for the period 2002–2014	According to the results of the study, factors such as age, marital status, disability status, income, gender, access to health services, household size, employment, education, place of residence and insurance status have an impact on the likelihood of catastrophic health expenditures.
Aregbeshola and Khan (36)	Nigeria	Regression	2010 data from the Harmonized Nigeria Living Standards Survey	The total expenditure, age, education level, chronic disease status, and health insurance status of the household are among the factors that increase the risk of catastrophic health expenditure.
Barkat et al. (41)	Arab countries	Co-integration	Data for 18 Arab countries for the period 1995–2015	Income is not the only determinant of health. Aging population and medical advances play an important role in increasing health expenditures.
Bayar et al. (46)	EU countries	Co-integration	Data for 27 EU countries for 2000–2018	According to the study results, real GDP per capita and life expectancy have a significant positive effect on health expenditures.
Yetim et al. (52)	OECD	Panel data analysis	Data for OECD countries for the period 2000–2017	The most significant factors affecting health expenditures were found to be income and education.
Adjei-Mantey and Horioka (37)	Ghana	Regression	Household survey data	The availability of health facilities significantly reduces health expenditures.
Piscopo et al. (34)	EU countries	Regression	Data for EU countries for the period 2000–2018	The increase in GDP has an impact on differences in per capita public health expenditure.

Herwartz and Theilen investigated the determinants of health expenditures (48). Barkat et al. analyzed the long and short-run determinants of health (41). Sinha et al. focused on the factors contributing to catastrophic health expenditures and out-of-pocket payments (32). Similarly, Folahan and Awe, as well as Hitiris and Posnett, investigated key drivers of health expenditure (43, 49). In a subsequent study, Herwartz and Theilen further examined these determinants (47). Martín et al. conducted a comprehensive literature review on the topic (57), while Hosoya assessed structural factors influencing health expenditures (53). Bayar et al. evaluated the effects of life expectancy, environmental factors, and real GDP per capita on per capita health spending (46). Wang, Samadi and Rad, and Cantarero and Lago-Peñas each contributed additional empirical insights into the determinants of health expenditures (28, 29, 42).

Studies in the literature on the determinants of health expenditures have been conducted in numerous countries. Accordingly, studies on this subject have been conducted in Türkiye (39, 54), OECD countries (19, 47, 52, 53, 56), Africa (30, 33), Nigeria (36, 43, 50), Malaysia (40, 44), in Ghana (37, 45), Lesotho (31), Spain (58), African countries (55), Association of Southeast Asian Nations (38), Italian Regions (51), Pakistan (35), EU countries (34, 46), United States (29), Spanish regions (28), ECO (42), India (32) and Arab countries (41).

There are many studies in the literature on the determinants of health expenditures. These studies have been conducted in different countries and using different methods. Since these studies contain details about the health system of each country, the relevant studies have been analyzed in detail in order to enable policy and system comparisons across countries. In this regard, the study question was designed since there is no study in the literature on Türkiye’s health system after the pandemic (covid-19) and in line with the referral system. On the other hand, studies that focus on health expenditures in a different way have been addressed in the study since they may lead to different findings in addition to different health policies and systems.

Although the studies in the literature address health expenditures from different perspectives, they do not focus on the referrals in the process of individuals’ demand for health expenditures. Especially in countries like Türkiye, where referral system is not mandatory, researching this issue and filling this gap indicates that a positive result can be provided to the shaping of health expenditures. As a matter of fact, the initiatives in the health system regarding this issue and the recently launched “Routing to the Family Physician” (26) practice support the aforementioned points. Accordingly, it is important to evaluate the factors affecting the decisions on health expenditures and to identify the determinants of health expenditures.

As determinants of health expenditures in the literature income (28, 40, 52, 58, 59), technological changes (56, 60), aging population (29, 41, 47, 56, 57), medical advancements (41), households (50), the ‘gatekeeping role’ of physicians (19), non-income factors (30), number of physicians (31, 42, 43), number of nurses (43), number of hospitals (43, 54), disadvantaged groups (32), out-of-pocket payments (44), urbanization (33, 42), technology and income elasticity (48), inflation, birth rate and life expectancy (45), GDP (34, 42, 49), the population under the age of 15 (42), the population over the age of 65 (29, 40, 42), unemployment (51, 53), time, ratio of the female population aged 15–64 to female labor force, persons per square kilometer (53), price (40), GDP per capita (35, 53, 54), life expectancy at birth (54), child immunization and female literacy (31), proximity to death (57), total expenditure, age, chronic disease status (36), health insurance status (36, 56), population per health personnel, official development assistance and decreases in income inequality (55), availability of health facilities (37), increasing volume of industrialization and foreign investment (38), education (36, 51, 52), urbanization (29, 35, 51), number of beds (29, 51), real Gross State Product (51), literacy rate, foreign aid and crude birth rate (35), is the subject.

This study’s focus on healthcare expenditure in relation to referral system policy and its examination of recently implemented practices (such as the Approved Appointment period and Routing to Family Doctors) are among its strengths. Another strength is its consideration of the post-pandemic period and its examination of differences in individuals’ levels of health literacy.

## Methods

### Data source

In this study, the micro data set of the Household Budget Survey conducted by the Turkish Statistical Institute (TurkStat) in 2022 is used. All settlements within the borders of the Republic of Türkiye constitute the geographical scope of the survey. The basic sampling framework used in the selection of blocks, which are the first stage sampling units in the 2022 Household Budget Survey, is the National Address Database. Blocks were created using this framework, blocks were determined from urban areas and rural areas with municipal organizations and villages with a probability proportional to the size of the settlement, and households were systematically selected from each block. The household located at the sample address was defined as the final sampling unit. The stratified two-stage cluster sampling method was used. Stratified two-stage cluster was used as the sampling method. The 2022 Household Budget Survey was conducted between January 1 and December 31, 2022, and the number of valid households was 11.922. The sampling structure of the survey was created in accordance with the purpose of providing estimates on the basis of “Türkiye” (61). Thus, 11.922 heads of households were included in the study.

### Determinants

#### Outcome variable

The outcome variable of the study is whether households make out-of-pocket health expenditures or not. Out-of-pocket health expenditures are expenditures that are not covered by the state or social security institutions and are made directly from individuals’ own budgets. In this study, health expenditures include expenditures on medicines, health products, therapeutic tools and materials (eyeglasses, lenses, neck braces, hearing aids, walkers, etc.), health-related tools (sphygmomanometers, glucometers, etc.), hospital and non-hospital medical services (doctor’s examination fee, dental services, X-ray, ultrasound, tomography, analysis, etc.), hospital beds, surgery, delivery, physical therapy, ambulance, etc. The presence of health expenditures was coded as 1 and the absence of health expenditures was coded as 0.

#### Explanatory variables

The study evaluated explanatory variables from two distinct perspectives. These are indicators for heads of household and household indicators. Indicators for heads of household can be listed as gender, age, education level, and employment status. Household indicators can be listed as the number of people in the household, private insurance ownership (whether the household has a member with private life insurance), income quartiles (1st quartile, 2nd quartile, 3rd quartile, 4th quartile), receipt of monetary aid (financial assistance given to the household in cash from spouses, friends, relatives or other non-relatives and households), household food expenditure value, access to health services (having easy access to “health center” services based on the location of the residence), alcohol use (whether there is a household member who consumes alcohol) and tobacco use (whether there is a household member who consumes tobacco and its products). Table 2 provides additional information on the explanatory variables.

**Table 2:** Explanatory variables.

Variables	Categories					
Individual-Level Indicators						
Gender of the head of household	Male	Female				
Age of the head of household	15–24	25–34	35–44	45–54	55–64	65 and over
Education level of the head of household	Did not graduate	Primary school graduate	High school graduate	High school, undergraduate-graduate degree		
Employment status of the head of household	Employed	Unemployed				
Household Indicators						
Number of persons in the household						
Presence of household members with private life insurance	Yes	No				
Annual household income quartile	1st quartile	2nd quartile	3rd quartile	4th quartile		
Household food expenditure	TL					
Household education expenditure	TL					
Receipt of monetary aid	No	Yes				
Difficulty of access to “Health Center” services based on the location of the household	Easy	Difficult				
Presence of household members who have a habit of drinking alcoholic beverages	Yes	No				
Presence of household members who smoke cigarettes, tobacco, or cigars	Yes	No				

Although the variables used in the study are generally related to health status and services, variables that can be considered different (alcohol and tobacco use, etc.) were also analyzed in the study. The reason for this is to provide a perspective on the effectiveness of Türkiye’s health policies toward addictions. In Türkiye’s health system, there are health policies that prevent and treat individuals’ addictions, and it is aimed to provide the necessary services through primary health care institutions and various non-governmental organizations (Green Crescent, etc.). Alcohol and tobacco addiction of individuals has a negative impact on their health and the health of those around them. This situation increases health expenditures by forming the basis for increased treatment and thus the need for health services. Therefore, by analyzing the relevant variables in the study, it is aimed to evaluate policies on addictions and to make various recommendations. Table 2 provides additional information on the explanatory variables.

### Empirical strategy

First, frequency analyses were conducted to determine whether households in the study incurred out-of-pocket health expenditures. Then, the factors influencing these expenditures and the effect sizes of these factors were determined using a binary probit regression model. Stata 16 (Stata Corporation) and Microsoft Excel were used to organize the dataset and perform statistical analyses. In this study, discrete choice model was applied to analyze the factors affecting the probability of households making out-of-pocket health expenditures. Consistent with the binary structure of the dependent variable, both the lbinary ogit model and the binary probit model were established with the same set of independent variables. Because the probit model generally exhibits a better model fit, the binary probit model was used in the study. The binary probit model is frequently used to model the probability of reaching an outcome when the dependent variable consists of only two categories (e.g., made/did not make health expenditures). This model is based on the assumption that there is a “latent” utility function underlying the tendency to choose a particular outcome (62). The explanatory variables used in the model were selected from the indicators stated in the literature to be related to access to health services and spending behaviors and included in the survey.

The basic hypotheses tested within the scope of the research are as follows:

*H*_1_: There is a significant relationship between the Age variable and the status of making out-of-pocket health expenditures.

*H*_2_: There is a significant relationship between the Gender variable and the status of making out-of-pocket health expenditures.

*H*_3_: There is a significant relationship between the Educational Status variable and the status of making out-of-pocket health expenditures.

*H*_4_: There is a significant relationship between the Marital Status variable and the status of making out-of-pocket health expenditures.

*H*_5_: There is a significant relationship between the Employment Status variable and the status of making out-of-pocket health expenditures.

*H*_6_: There is a significant relationship between the Private Insurance Ownership variable and the status of making out-of-pocket health expenditures.

*H*_7_: There is a significant relationship between the Income Quartile variable and the status of making out-of-pocket health expenditures.

*H*_8_: There is a significant relationship between the Receipt of Monetary Aid variable and the status of making out-of-pocket health expenditures.

*H*_9_: There is a significant relationship between the Access to Health Center variable and the status of making out-of-pocket health expenditures.

*H*_10_: There is a significant relationship between the Alcohol Use variable and the status of making out-of-pocket health expenditures.

*H*_11_: There is a significant relationship between the Tobacco Use variable and the status of making out-of-pocket health expenditures.

**Figure 2:**
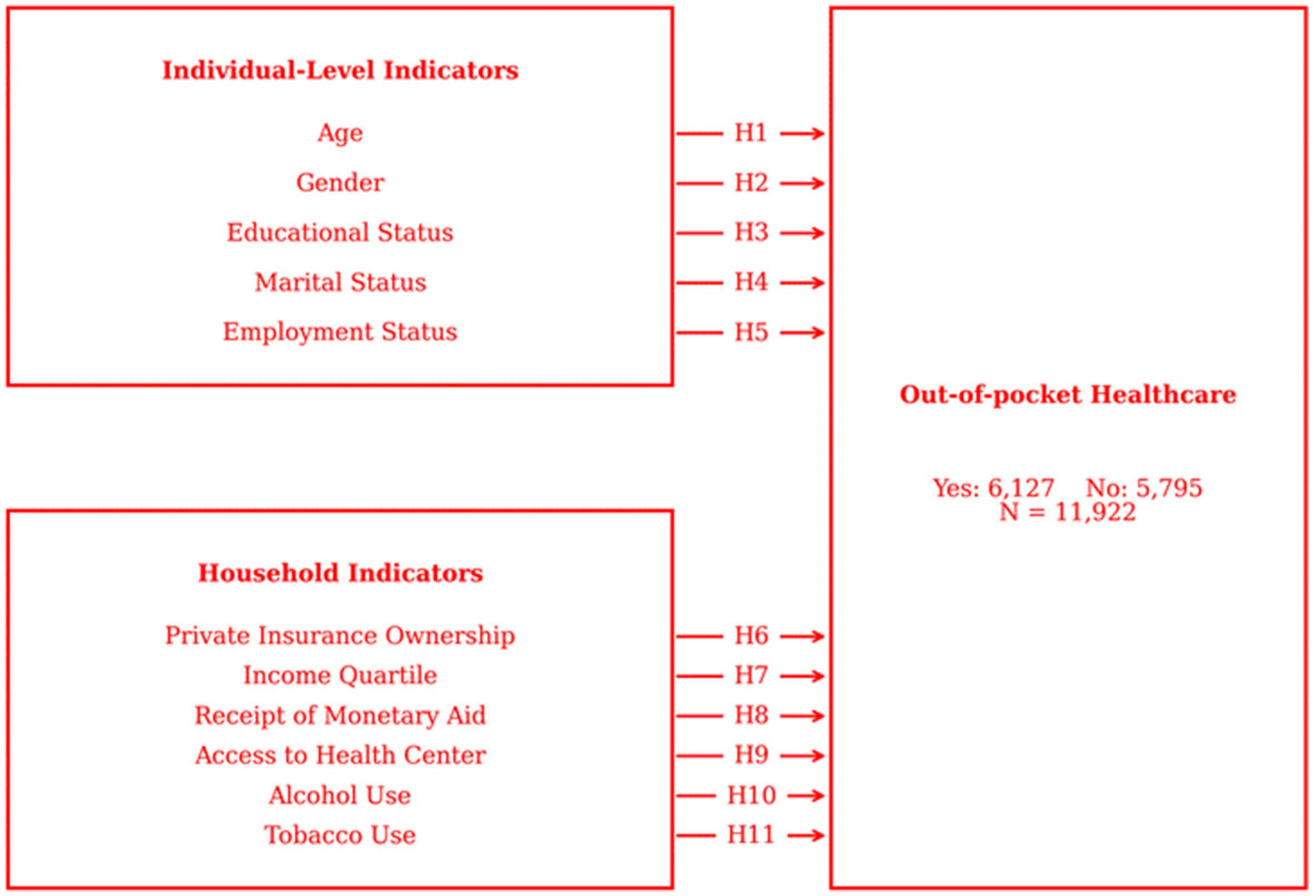
Research model.

Figure 2 illustrates how the hypotheses analyzed in the study are structured according to the theoretical framework. The effects of individual-level variables (e.g., age, gender, education level) and household-level variables (e.g., household size, income satisfaction, household structure) on the likelihood of spending on health care are represented by directional arrows in the conceptual model. Each arrow represents the corresponding hypothesis (H1–H11).

## Results

### Characteristics of study participants

Tables 3, 4 show the descriptive statistics regarding the factors thought to be associated with out-of-pocket health expenditures.

**Table 3:** Descriptive statistics on out-of-pocket health expenditures.

Variables	Status of making out-of-pocket health expenditures		n(%)
	No	Yes	
Individual-Level Indicators			
*Gender*			
Male	75.10%	75.30%	75.20%
Female	24.90%	24.70%	24.80%
*Age*			
15–24	1.70%	1.30%	1.50%
25–34	13.30%	12.20%	12.70%
35–44	22.40%	21.70%	22.10%
45–54	22.20%	22.80%	22.50%
55–64	20.40%	20.70%	20.50%
65+	20.10%	21.30%	20.70%
*Educational Status*			
Did Not Graduate	11.50%	11.90%	11.70%
Primary Education Graduate	51.20%	53.50%	52.30%
High School Graduate	19.20%	17.50%	18.30%
Undergraduate-Graduate, Post-Graduate Degree	18.20%	17.10%	17.60%
*Employment Status*			
Employed	61.30%	60.50%	60.90%
Unemployed	38.70%	39.50%	39.10%
Household Indicators			
*Private Insurance Ownership*			
Yes	10.50%	9.50%	10.00%
No	89.50%	90.50%	90.00%
*Income Quartile*			
1st quartile	23.80%	26.10%	25.00%
2nd quartile	24.40%	25.60%	25.00%
3rd quartile	26.30%	23.80%	25.00%
4th quartile	25.50%	24.50%	25.00%
*Receipt of Monetary Aid*			
No	88.50%	89.10%	88.80%
Yes	11.50%	10.90%	11.20%
*Access to Health Center*			
Easy	71.50%	70.10%	70.80%
Difficult	28.50%	29.90%	29.20%
*Alcohol Use*			
Yes	7.20%	6.80%	7.00%
No	92.80%	93.20%	93.00%
*Tobacco Use*			
Yes	51.80%	52.60%	52.20%
No	48.20%	47.40%	47.80%

**Table 4:** Descriptive statistics.

Variable	Obs	Mean	Std. Dev.	Min	Max
Food Expenditure	11.922	2869.286	2113.590	0	35,774
Education Expenditure	11.922	198.781	1025.908	0	41679.170
Number of Persons in the Household	11.922	3.287	1.661	1	18

The current structure of the households was revealed through descriptive statistics. According to the tables, the average household size of Turkish households is 3.29 persons. The average monthly food expenditure is 2869.29 TL. 75.1% of heads of households are male, 22.4% are between the ages of 35–44, and 51.2% are primary school graduates. Only 10.5% of households have private life insurance, 11.5% receive monetary aid, and 28.5% have difficult access to health centers due to their location.

### Probit model results

A binary probit regression model was used to determine the attitudes of the households in the study toward out-of-pocket health expenditures. The model was found to be statistically significant (*p* < 0.0001). Table 5 shows the model estimation results and variance inflation factor values of the explanatory variables.

**Table 5:** Binary probit regression model and variance inflation factors.

Değişkenler			Coef.	Std. Err.	Prob.	Vif
Individual-Level Indicators						
Gender	Male (reference category)					
	Female		−0.011	0.031	0.717	1.37
Age	15–24		−0.196	0.103	0.057	1.14
	25–34		−0.098	0.050	0.048	2.06
	35–44		−0.052	0.045	0.244	2.61
	45–54		−0.016	0.042	0.695	2.31
	55–64		−0.034	0.038	0.368	1.77
	65 + (reference category)					
Educational Status	Did Not Graduate (Reference category)					
	Primary Education Graduate		0.026	0.041	0.524	3.22
	High School Graduate		−0.025	0.050	0.615	2.86
	Undergraduate-Graduate, Post-Graduate Degree		0.013	0.054	0.807	3.19
Employment Status	Employed		0.019	0.032	0.551	1.78
	Unemployed (Reference category)					
Household Indicators						
Number of Persons in the Household			−0.005	0.008	0.558	1.38
Private Insurance Ownership	Yes		−0.037	0.041	0.366	1.14
	No (Reference category)					
Income Quartile	1st quartile (Reference category)					
	2nd quartile		−0.037	0.034	0.275	1.66
	3rd quartile		−0.127	0.036	0.000	1.84
	4th quartile		−0.094	0.039	0.017	2.18
Food Expenditure			0.000	0.000	0.000	1.02
Education Expenditure			0.000	0.000	0.000	1.01
Receipt of Monetary Aid	No		−0.035	0.038	0.352	1.09
	Yes (Reference category)					
Access to Health Center	Easy (Reference category)					
	Difficult		0.015	0.027	0.581	1.10
Alcohol Use	Yes		−0.015	0.047	0.742	1.07
	No (Reference category)					
Tobacco Use	Yes		0.037	0.024	0.129	1.11
	No (Reference category)					
McFadden’s R^2^	0.011	Number of obs	11,922			
Cragg & Uhler’s R^2^	0.020	Prob > chi2	0.000			
McKelvey & Zavoina’s R^2^	0.026	Log likelihood	8175.9748			

Whether the explanatory variables included in the model carried multicollinearity problems was checked with variance inflation factors (VIF). While VIF values between 5 and 10 indicate a moderate multicollinearity problem, values smaller than 5 show no multicollinearity problem (63). VIF values below 5 indicate that there is no multicollinearity.

Due to the nature of discrete choice models, quantitative interpretations of the coefficients will be made through marginal effects. Table 6 shows the marginal effects obtained from the model estimation.

**Table 6:** Marginal effect estimation results.

Variables	ey/dx	Std. Err.	Prob.	95% Conf. interval	
				Lower limit	Upper limit
Individual-Level Indicators					
*Gender*					
Male (Reference category)					
Female	−0.009	0.024	0.717	−0.057	0.039
*Age*					
15–24	−0.160	0.089	0.074	−0.335	0.015
25–34	−0.077	0.039	0.049	−0.154	0.000
35–44	−0.040	0.035	0.243	−0.108	0.027
45–54	−0.012	0.032	0.695	−0.075	0.050
55–64	−0.026	0.029	0.367	−0.083	0.031
65 + (Reference category)					
*Educational Status*					
Did Not Graduate (Reference category)					
Primary Education Graduate	0.021	0.032	0.527	−0.043	0.084
High School Graduate	−0.020	0.040	0.614	−0.098	0.058
Undergraduate-Graduate, Post-Graduate Degree	0.010	0.042	0.807	−0.072	0.093
*Employment Status*					
Employed	0.015	0.025	0.551	−0.034	0.063
Unemployed (Reference category)					
Household Indicators					
Number of Persons in the Household	−0.004	0.006	0.558	−0.016	0.009
*Private Insurance Ownership*					
Yes	−0.029	0.033	0.372	−0.093	0.035
No (Reference category)					
*Income Quartile*					
1st quartile (Reference category)					
2nd quartile	−0.028	0.026	0.275	−0.078	0.022
3rd quartile	−0.099	0.028	0.000	−0.153	−0.044
4th quartile	−0.072	0.030	0.017	−0.131	−0.013
Food Expenditure	0.000	0.000	0.000	0.000	0.000
Education Expenditure	0.000	0.000	0.000	0.000	0.000
*Receipt of Monetary Aid*					
No	−0.028	0.030	0.357	−0.087	0.032
Yes (Reference category)					
*Access to Health Center*					
Easy (Reference category)					
Difficult	0.011	0.021	0.580	−0.029	0.052
*Alcohol Use*					
Yes	−0.012	0.037	0.743	−0.084	0.060
No (Reference category)					
*Tobacco Use*					
Yes	0.029	0.019	0.129	−0.008	0.066
No (Reference category)					

Based on the marginal effects shown in Table 5, when out-of-pocket health expenditures are analyzed in terms of the head of the household, the gender, education level and employment status of the head of household have no statistically significant effect on out-of-pocket health expenditures, while the age of the head of household being between 15–24 and 25–34 years decreases the probability of out-of-pocket health expenditures by 16 and 7.7% compared to the reference group. Regarding household-level variables, private insurance ownership, alcohol and cigarette use, easy access to health centers due to the location of the household, receiving monetary aid, and household size have no statistically significant effect on out-of-pocket health expenditures. On the other hand, an increase in food and education expenditures increases the probability of out-of-pocket health expenditures and the probability of out-of-pocket health expenditures of households in the third and fourth income quartiles is 9.9 and 7.2% lower, respectively, compared to the reference group.

## Discussion

The present study examines the factors influencing out-of-pocket health expenditures among Turkish households. The data set encompasses both readily available variables and variables constructed by the researchers based on the data set. Out-of-pocket health expenditures are analyzed in two dimensions: indicators for the head of the household and household indicators. The data set is representative of Türkiye and consists of official institutional data.

The analysis revealed no statistically significant correlation between the gender of the head of the household and out-of-pocket health expenditures. In contrast to this finding, studies within the existing literature (39) have indicated that gender is a significant factor influencing the probability of incurring catastrophic health expenditures. While social roles may vary between men and women, it is generally accepted that healthcare is a fundamental need for all individuals regardless of gender. Nevertheless, the influence of literacy levels on health and health services and their effect on the demand for health care is well established. In this regard, it is thought that this finding, which is divergent from the results of a previous study conducted in 2017 (39), may be associated with the advancement of technology, the rising health literacy rate, the globalization of society, and the pandemic.

Heads of household in the 25–34 age range were found to be less likely to make out-of-pocket health expenditures compared to those aged 65 and over, and heads of household in the 15–24 age range were even less likely to make out-of-pocket health expenditures compared to those aged 65 and over. Similarly, it has been found that the degree of old age is the main determinant of health expenditures (29, 41); health expenditures become more volatile with aging (47); the proportion of the population over 65 is among the important variables in terms of health expenditures (40); the aging population is a common factor in the determinants of health expenditures (57); there is a short-run relationship between the ratio of population under 15 and over 65 and health expenditures (42); the ratio of the female population aged 15–64 to the female labor force has an impact on health expenditure (53); aging of the population is among the main factors that increase the cost of health services (56); and age has an effect on the probability of catastrophic health expenditures (36, 39). The findings of the present study are in line with the results of the studies in the literature. Age is an important factor in the increasing need for health. In this direction, considering that the demand for health services will increase as individuals age, there are policies designed for this purpose in the health system. The follow-up of chronic diseases in primary health care services for the older adult(s) (family health centers) can be evaluated in this context. Therefore, it is predicted that the older adult(s) will spend more on health expenditures, and health policies for the older adult(s) are designed accordingly. The findings of the study support this prediction.

It was determined that there was no statistically significant correlation between the education level of the heads of household and out-of-pocket health expenditures. However, existing literature reports that literacy rate is one of the determinants of health expenditures (35); female literacy is among the important determinants of health expenditures (31); the percentage of the population with at least secondary school education has a direct impact on health expenditures (51); education has an impact on the probability of catastrophic health expenditures (36, 39); and education is among the most important factors affecting health expenditures (52). Although the education variable is an important factor in shaping health expenditures, its positive influence depends on the level attained. Notably, individuals with low education levels question the health services they receive more. This trend is further reflected in the increasing number of reported incidents of violence in healthcare settings. On the other hand, low education levels may result from low income levels. In Türkiye’s health system, households with a monthly income of less than one-third of the minimum wage previously received healthcare coverage through the “Green Card” program and are now covered by “General Health Insurance.” The lack of a significant correlation between education level and health expenditures may thus reflect these policy developments and socioeconomic factors.

There is no statistically significant correlation between the employment status of the heads of household and out-of-pocket health expenditures. However, several studies suggest that employment status is an important factor affecting health expenditures, with both unemployment (53) and employment (39) influencing the likelihood of catastrophic health spending. It is thought that the difference between the findings of this study and the literature may be due to differences in study scope or context. The employment status, and thus monthly income, of individuals may affect health financing according to the structure of their country’s health system. While this situation can be monitored based on the occupational and social status of individuals in France (27), it is similarly provided by the “General Health Insurance” in Türkiye. In this context, national health financing policies and systems are thought play a crucial role. After analyzing the relationship between the study variables and household health expenditures, indicators related to households were also analyzed. Accordingly, the effects of education expenditures, income level, private insurance coverage, alcohol and tobacco use, access to health centers, receipt of monetary aid, and household size on health expenditures were also examined.

The probability of out-of-pocket health expenditures increased as education expenditures increased. Several studies have identified literacy rate (35), female literacy (31), attainment of at least secondary education (51), and overall education level (36) as key determinants of health expenditures and as factors that can increase the likelihood of such expenditures. Higher education expenditures result in an improved level of education, which may, in turn, lead to more cautious and informed health service utilization. It is expected that the increase in education expenditures will also increase health expenditures. People with higher levels of education are most likely to be aware of the updates in Türkiye’s health system, changing regulations and developments such as digital hospitals. This increases the likelihood that these individuals will benefit more from the relevant services and thus increase the likelihood of health expenditures. The finding of the study, which is consistent with the literature, supports this idea.

An increase in income level decreases the likelihood of out-of-pocket health expenditures. Previous studies have identified income elasticity (48), reductions in income inequality (55), foreign aid (35), and income levels (28, 32, 39, 40, 44, 52, 58) as determinants of health expenditures, with these factors generally being positively correlated with an increase in such expenditures. Since this study was conducted in Türkiye during 2022, the period immediately following the pandemic, it is likely that individuals have become more selective in their use of health services. In this context, individuals with higher income levels may choose to seek health services abroad through health tourism, and the findings of this study may reflect the impact of these trends. The recent global pandemic (COVID-19) is expected to have an impact on health tourism as in all health services. Indeed, Türkiye’s health tourism data (756,926 patients in 2019, 435,691 patients in 2020, 729,592 patients in 2021, 1,381,807 patients in 2022, 1,538,643 patients in 2023, 1,506,442 patients in 2024) support this prediction (4). In particular, the fact that the number of patients receiving services from Türkiye within the scope of health tourism continues to increase every year, while this number decreased in 2020 and then continued to increase, is an indication of the determining role of the pandemic on health tourism and that people are taking initiatives to seek better health services. It is thought that the finding obtained may be due to these factors.

There is no statistically significant correlation between the private insurance coverage of household members and out-of-pocket health expenditures. Previous studies have examined the relationship between social security, insurance, and health expenditures. These studies have identified health service prices (40), the presence of insurance (39, 56, 64), and health insurance status (36) as important variables influencing health expenditures, which can raise both the cost of services and the likelihood of incurring health-related expenses. In Türkiye’s health financing method (General Health Insurance), the insured status of individuals is based on certain variables (gender, age, education, etc.). In this sense, women, dependents and single individuals, regardless of their educational status, can benefit from the premiums of their family members in health financing. On the other hand, men’s premiums are financed by the state even if they do not exceed the age of 20 for high school and equivalent schools and 25 for higher education (65). The premiums of those who do not meet the specified conditions are covered by the state for a certain period of time (2 years), while in the following period, individuals are indebted for certain premium payments for each month. As of January 2025, the premium amount required for individuals to benefit from health insurance was determined as 780.17 TL per month (66). Therefore, it is predicted that people who do not qualify for health insurance premium payment may be more inclined to pay for private health insurance, which allows different opportunities in public and private health institutions. Accordingly, individuals with private or complementary health insurance are expected to spend more on health expenditures. However, similar to the fact that hospitals were historically avoided due to epidemics, people today remain cautious about seeking hospital care for various reasons, including lingering effects of the pandemic. Furthermore, the COVID-19 pandemic, particularly during the quarantine period, encouraged individuals to manage their own health, thereby increasing their health literacy levels. Therefore, the idea that the more hospital admissions, the better, has been discarded in recent years, and this perspective has contributed to reducing health expenditures, as observed in this study. It is thought that this finding may be related to the aforementioned issues. There is no statistically significant correlation between out-of-pocket health expenditures and alcohol and tobacco use by household members. In contrast to the findings of this study, previous studies have indicated that proximity to death (57) and life expectancy (45, 46) are important determinants of health expenditures, both having a positive effect on such expenditures.

There is no statistically significant correlation between the location of the household and easy access to health centers and out-of-pocket health expenditures. The main reason for including physical access to health services among the study variables is to evaluate Türkiye’s policies on the subject and to provide a perspective in this sense. In order to remove the barriers to physical access, the number of health institutions is increasing day by day and primary health care institutions (family physicians, family health centers, etc.) are positioned in a pedestrian accessible way in line with certain regulations (unsanitary establishments regulation, etc.). Therefore, it is thought that various suggestions can be made for policy makers by examining the role of Türkiye’s health policies, which aim to remove the barriers to physical access, on expenditures, and the relevant variable is included in the study. Previous studies have reported a positive relationship between urbanization (29, 33, 35, 42, 45), population density (53), access to health services (39), availability of health facilities (37), and health expenditures, identifying these factors as important determinants that increase health spending. The discrepancy between this study’s findings and the existing literature may be attributed to differences in the health systems of the countries where the studies were conducted. While referral systems are widespread in many countries’ health systems (and sometimes even mandatory) in Türkiye, family health centers play a significant role in facilitating access to health services. However, since there is no requirement to visit family health centers (the gatekeeper role of family physicians) in Türkiye, these centers may be perceived as less important by individuals and households. Therefore, patients who can easily access the health institution of their choice are unlikely to attach significant importance to easy access to health centers when it comes to their expenditures. Based on the findings of the study, it can be said that the expectation regarding Türkiye’s health system is supported.

There is no statistically significant correlation between the receipt of monetary aid and out-of-pocket health expenditures. However, previous studies have shown that foreign aid (35) and income (28, 39, 40, 44, 52, 58) are important determinants of health expenditures, and there is often a relationship between these variables and health spending. Households/individuals in need of monetary assistance are categorized as households/individuals whose monthly income is less than 1/3 of the minimum wage in the “General Health Insurance.” The finding in the present study, which contradicts the literature, may be related to Türkiye’s policies on health financing. There is no statistically significant correlation between household size and out-of-pocket health expenditures. Studies in the literature have identified crude birth rate (35), individual demographic factors (50), and household size (39) as determinants of health expenditures, which may influence the likelihood and amount of health-related spending. As mentioned above, crowded households may have low income levels, which may allow them to receive monetary aid and have their health services financed by the state.

## Conclusion

The necessity of health and health services, and the inability to substitute or defer them, underpins the rationale for determining the size of health expenditures and their continued growth. In this regard, a number of studies have been conducted at the global level with the objective of developing an optimal approach to the management of health expenditures. While the overarching objective of these studies is to identify optimal strategies for managing healthcare expenditures, the specific approaches employed may vary across different countries. In this study on the determinants of health expenditures in Türkiye, the findings will be interpreted in light of the country’s strategy, policy, and system for health expenditures.

In this study, the determinants of health expenditures are identified using a discrete choice model, based on the 2022 Household Budget Survey microdata from the Turkish Statistical Institute (TurkStat). The findings of this study diverge from those of the existing literature. It is hypothesized that this situation may be related to the recent global pandemic (COVID-19), and that the pandemic has opened the door to differences in individuals’ health service procurement and played an important role in differentiating their expenditures.

The findings of the study indicate that individuals in the younger and middle age groups tend to allocate a smaller proportion of their expenditure to healthcare than those aged 65 and above. Life expectancy at birth represents a significant global indicator of health status. As a result, many countries are implementing initiatives aimed at increasing life expectancy and extending the overall lifespan of their populations. In Türkiye, older adult(s) individuals are monitored for a range of diseases in health institutions, primarily family health centers, with the objective of improving their health status. Türkiye’s favorable performance in key health indicators not only strengthens its national healthcare outcomes but also contributes to its international reputation. Furthermore, improvements in health metrics have helped position Türkiye as a preferred destination for health tourism. Conversely, an analysis of Türkiye’s historical health policies in relation to health expenditure indicates that the General Health Insurance, health literacy studies, follow-up of specific diseases in the older adult(s) by family physicians and the Approved Appointment period have been pivotal in reducing the overall health expenditure of individuals. It is therefore anticipated that the health expenditure of the older adult(s) will increase in light of the aforementioned circumstances. Indeed, the findings of the study support this expectation.

In contrast to the assertion that there is no significant correlation between the education variable and health expenditures, the present findings suggest that there is a positive relationship between education expenditures, which are considered within the scope of household indicators, and health expenditures. The level of education clearly plays a pivotal role in the process of demand for health services. Notably, during the COVID-19 pandemic, individuals had to navigate health services independently, which encouraged them to conduct research and develop their understanding of available health resources. This situation contributes to an increase in the level of health literacy and a healthier management of the health service process. As a result, individuals are now better equipped to select the most appropriate physicians, institutions, and locations for their healthcare needs. This indicates that the rise in educational attainment resulting from increased educational spending may exert a significant influence on the formation of health expenditure patterns. Conversely, the absence of a notable correlation between the education variable and health expenditures in the study may be attributed to the relatively low level of education. The referral system in Türkiye also plays a role in these findings. Unlike some other countries, the Turkish referral system does not give family physicians a mandatory gatekeeper role, which may suggest that the intended policy goal has not been fully realized. Indeed, the findings obtained for the variables of education and health expenditures suggest that assigning a gatekeeper role to family physicians in primary health care services will result in positive outcomes in terms of health expenditures. Therefore, it can be said that increasing education expenditures create a healthy service process and this situation plays a role in shaping health expenditures.

The results of the study indicate that there is no statistically significant correlation between health expenditures and the variables of gender, education level, employment status, private insurance ownership, alcohol and tobacco use, proximity of the household to health centers, receipt of monetary aid and household size. Due to the essential nature of health services and the fact that their use cannot be postponed, financial considerations may not significantly influence access to or use of these services. In terms of the health system, the constitutional support of health services as a human right and a duty of the state has historically necessitated the involvement of third parties in the financing of health services. To achieve this, multiple third-party institutions (such as SSK, Bağ-Kur, the Pension Fund, and the Green Card program) have historically formed part of the Turkish health system, each providing different forms of social security. It is worth noting that the General Health Insurance, which was introduced in 2012 as part of the Health Transformation Program, is still in place today. In the Turkish health system, expenditure on health services may vary depending on the level of service provision and the insurance category of the individual. However, although there are differences in expenditures, the provision of and demand for these services generally occur without regard to individuals’ socio-demographic or socio-economic characteristics. Therefore, it can be said that the lack of a significant relationship between health expenditure and related variables may be related to the aforementioned issues.

The present study has determined that an increase in income level, as evaluated in terms of indicators pertaining to households, is associated with a reduction in expenditure on health. Historically, individuals often avoided hospitals (particularly during epidemics) and instead preferred home treatment. Consequently, hospitals were utilized by homeless individuals during the specified period. In the subsequent period, hospitals became the preferred option due to the prevention of epidemics, the high cost of home treatment and the difficulty in obtaining large devices (such as tomography equipment) used in the treatment process at home. While a number of factors may have contributed to this shift in preference, the prevalence of epidemics is likely to have been a significant driver. It can be deduced that those with high income levels may opt for home care. Indeed, a comparable scenario is currently unfolding, wherein the notion that superior healthcare can be accessed in the home, at a private clinic, or in a private hospital may once again gain traction. This trend may be related to the COVID-19 pandemic, which has influenced healthcare behaviors in ways similar to previous historical events. Therefore, it can be posited that the current situation can be more readily understood with the results of the recently initiated “Health Literacy” survey by the Ministry of Health, which could inform the development of new policies on income and health expenditures in light of the findings of this study.

Based on the findings of this study, the following policy recommendations can be made:

- The relationship observed between education and health expenditures highlights the crucial role of health literacy. The implementation of comprehensive studies on health literacy at the regional, provincial, and district levels will facilitate the management and direction of the health service process and thus expenditures in a manner that aligns with desired outcomes. The Turkish Ministry of Health’s Directorate General for Health Promotion conducted the “Study on Health Literacy Levels and Related Factors in Türkiye” in 2018 (67) and 2024 (68). The study provides a general profile of Türkiye. A similar study could be conducted by the General Directorate of Health Promotion at the regional, provincial, and district levels, offering a more transparent perspective.
- It is hypothesized that the rise in income levels may be associated with a reduction in health expenditures and a shift in individuals’ preferences toward alternative health sectors, particularly health tourism. Therefore, it is thought that there may be a growing sense of distrust toward the health system. Based on the findings of relevant studies, policies designed to increase the confidence of people residing in Türkiye in the healthcare system can be formulated in such a way that individuals and households with high income levels demand more from Türkiye’s healthcare services. In this regard, when examining Türkiye’s health tourism data, (756,926 patients in 2019, 435,691 patients in 2020, 729,592 patients in 2021, 1,381, 807 patients in 2022, 1,538,643 patients in 2023, and 1,506,442 patients in 2024) (4), it is evident that there is global confidence in the system. Similarly designed policies at the national level indicate that high-income individuals will direct their healthcare demands toward Türkiye’s healthcare system.
- Based on the results obtained from the study, this study, which emphasizes the role of individuals in shaping health expenditures, supports that establishing a mandatory referral system will play a role in reducing expenditures. In this sense, it can be said that the gradual implementation of a mandatory referral system in Türkiye, in addition to increasing the number of health personnel, will allow time and cost savings. In this sense, the desired results in health expenditures can be achieved in line with the policies to be designed. During the preparation process of the study (July 25, 2025), the “Routing to Family Physicians” program (26) implemented by the Turkish Ministry of Health once again emphasized the importance of the above recommendation. As highlighted in the study, this situation not only points to the need for policy design for the referral system in health expenditures but also reflects the foresight and reality of the study’s findings.

## Data Availability

Publicly available datasets were analyzed in this study. This data can be found at: the data supporting the findings of this study are available from the Turkish Statistical Institute; however, access is restricted as the data were used under license for the present study and are not publicly available. Reasonable requests for data may be submitted to the authors with permission from the Turkish Statistical Institute.

## References

[1] Durgun F. Sağlık Harcamalarının Gelir Eşitsizliğine Etkisi: Türkiye’den Kanıtlar In: Uysal Şahin Ö ve Şeren GY, editors. Sağlık Ekonomisinde Paradigma Dönüşümü. Çanakkale: Holistence Publications (2023)

[2] Sey N. (2023) OECD ülkelerinde sağlık harcamalarının belirleyicileri üzerine ekonometrik bir analiz (Unpublished doctoral thesis). Istanbul University.

[3] Woodward RS Wang L. The oh-so straight and narrow path: can the health care expenditure curve be bent? Health Econ. (2012) 1:1023–9. doi: 10.1002/hec.1765 21755571

[4] USHAŞ. T.C. Sağlık Bakanlığı, Uluslararası Sağlık Hizmetleri A.Ş. Available online at: https://www.ushas.com.tr/saglik-turizmi-verileri/ (Accessed April 23, 2025).

[5] WHO. (2000). The world health report 2000: Health systems: Improving performance. Available online at: http://www.who.int/entity/whr/2000/en/whr00_en.pdf

[6] Gazete R. (1949). Türkiye Cumhuriyeti Emekli Sandığı Kanunu. Available online at: https://www.mevzuat.gov.tr/MevzuatMetin/1.3.5434.pdf (Accessed March 03, 2024).

[7] Yardim MS Cilingiroglu N Yardim N. Catastrophic health expenditure and impoverishment in Türkiye. Health Policy. (2010) 94:26–33. doi: 10.1016/j.healthpol.2009.08.006 19735960

[8] Tatar M Ozgen H Sahin B Belli P Berman P. Informal payments in the health sector: a case study from Türkiye. Health Aff. (2007) 26:1029–39. doi: 10.1377/hlthaff.26.4.1029 17630446

[9] Çiçeklioğlu M. (2011). Crisis of Capitalism and Health. Available online at: http://www.ttb.org.tr/kutuphane/kapitalizm.pdf (Accessed May 05, 2024).

[10] DPT. (1997). Devlet Planlama Teşkilatı. Available online at: www.dpt.gov.tr/DocObjects/Download/2985/saglik.pdf (Accessed August 06, 2024).

[11] Bakanlığı T. C. Sağlık. (2003). Sağlıkta Dönüşüm Programı. Available online at: https://www.saglik.gov.tr/TR,11415/saglikta-donusum-programi.html (Accessed March 03, 2024).

[12] Sosyal Güvenlik Kurumu Kanunu. (2006). Available online at: https://www.mevzuat.gov.tr/MevzuatMetin/1.5.5502-20140910.pdf (Accessed August 05, 2024).

[13] Akdağ R. Türkiye sağlıkta dönüşüm programı ilerleme raporu. Ankara: T.C. Sağlık Bakanlığı (2008).

[14] Sosyal Güvenlik Kurumu. (2024). Genel Sağlık Sigortası. Available online at: https://www.sgk.gov.tr/Content/Post/742c02df-68e1-422c-a387-fa2e4326b015/Genel-Saglik-Sigortasi-nedir-2023-01-25-11-25-46 (Accessed August 06, 2024).

[15] Aba G. Sağlık Politikası ve Planlanması. Ankara: Nobel Yayıncılık (2020).

[16] Üstü Y Uğurlu M Örnek M Sanisoğlu SY. 2002-2008 Yılları Arasında Erzurum Bölgesinde Birinci ve İkinci Basamak Sağlık Hizmetlerinin Değerlendirilmesi. Balkan Med J. (2011) 28:55–61.

[17] Kılınç AS Çatak B Badıllıoğlu O Sütlü S Dikme Ö Aydın O. Acil servise başvuran yaşlıların başvuru nedenlerinin ve sonuçlarının değerlendirilmesi. SDÜ Tıp Fakültesi Dergisi. (2012) 19:139–43.

[18] Rasoulynejad S. Study of self-referral fac tors in the three-level healthcare delivery system, Kashan, Iran, 2000. Rural Remote Health. (2004) 4:1–11. doi: 10.22605/RRH237 15887984

[19] Gerdtham UG Jönsson B MacFarlan M Oxley H. The determinants of health expenditure in the OECD countries: a pooled data analysis. Health. (1998) 6:113–34. 10.1007/978-1-4615-5681-7_6 10662400

[20] French S Old A Healy J. (2001). Health Care Systems in Transition new Zealand. Copenhagen: World Health Organization.

[21] Koçak E. (2014). OECD ülkelerin sağlık sistemlerine ilişkin etkinlik analizleri (Unpublished master’s thesis). Ankara University.

[22] Mossialos E Djordjevic A Osborn R Sarnak D. International profiles of health care systems. America: The Commonwealth Fund (2017).

[23] Özel B. (2024). SGK Özel Hastane Kararı. Available online at: https://www.hurriyet.com.tr/ekonomi/sgkdan-ozel-hastane-karari-42074664 (Accessed August 03, 2024).

[24] Çalık Göçümlü B. (2023) 15 binden fazla hanede “sağlık okuryazarlığı” araştırması başlatıldı aa.com.tr.

[25] Türkay Ş. (2024). Hastanelerde Onaylı Randevu Dönemi Başladı. Available online at: https://www.aa.com.tr/tr/saglik/hastanelerde-onayli-randevu-donemi-basladi/3217739 (Accessed August 03, 2024).

[26] Türker Yıldız D. (2025). MHRS’de Randevu Talebi Öncesi “Aile Hekimine Yönlendirme Uygulaması” Başladı. Available online at: https://www.aa.com.tr/tr/saglik/mhrsde-randevu-talebi-oncesi-aile-hekimine-yonlendirme-uygulamasi-basladi/3642753# (Accessed July 07, 2025).

[27] Chevreul K Zaleski ID Bahromi S Quevedo CH Mladovsky P. France: Health system review. Copenhagen: World Health Organization Publishing (2010).

[28] Cantarero D Lago-Penas S. The determinants of health care expenditure: a reexamination. Appl Econ Lett. (2010) 17:723–6. doi: 10.1080/13504850802314437

[29] Wang Z. The determinants of health expenditures: evidence from US state-level data. Appl Econ. (2009) 41:429–35. doi: 10.1080/00036840701704527

[30] Murthy VN Okunade AA. The core determinants of health expenditure in the African context: some econometric evidence for policy. Health Policy. (2009) 91:57–62. doi: 10.1016/j.healthpol.2008.10.001, PMID: 19108929

[31] Akinkugbe O Mohanoe M. Public health expenditure as a determinant of health status in Lesotho. Soc Work Public Health. (2009) 24:131–47. doi: 10.1080/19371910802569716, PMID: 19229779

[32] Sinha RK Chatterjee K Nair N Tripathy PK. Determinants of out-of-pocket and catastrophic health expenditure: a cross-sectional study. British J Med Med Res. (2016) 11:1–11. doi: 10.9734/BJMMR/2016/21470

[33] Gbesemete KP Gerdtham UG. Determinants of health care expenditure in Africa: a cross-sectional study. World Dev. (1992) 20:303–8.

[34] Piscopo J Groot W Pavlova M. Determinants of public health expenditure in the EU. PLoS One. (2024) 19:1–19. doi: 10.1371/journal.pone.0299359, PMID: 38446804 PMC10917289

[35] Toor IA Butt MS. Determinants of health care expenditure in Pakistan. Pak Econ Soc Rev. (2005) 43:133–50. doi: 10.22004/ag.econ.118422

[36] Aregbeshola BS Khan SM. Determinants of catastrophic health expenditure in Nigeria. Eur J Health Econ. (2018) 19:521–32. doi: 10.1007/s10198-017-0899-1, PMID: 28555372

[37] Adjei-Mantey K Horioka CY. Determinants of health insurance enrollment and health expenditure in Ghana: an empirical analysis. Rev Econ Househ. (2023) 21:1269–88. doi: 10.1007/s11150-022-09621-x

[38] Sagarik D. Determinants of health expenditures in ASEAN region: theory and evidence. Millenn Asia. (2016) 7:1–19. doi: 10.1177/0976399615624054

[39] Tokatlioglu Y Tokatlioglu İ. Türkiye’de Katastrofik Sağlık Harcamaları ve Bu Harcamaları Belirleyen Faktörler: 2002-2014 Dönemi. Sosyoekonomi. (2018) 26:59–78. doi: 10.17233/sosyoekonomi.302930

[40] Tang CF (2010). The determinants of health expenditure in Malaysia: A time series analysis. Munich personal RePEc archive. 24356.

[41] Barkat K Sbia R Maouchi Y. Empirical evidence on the long and short run determinants of health expenditure in the Arab world. Q Rev Econ Finance. (2019) 73:78–87. doi: 10.1016/j.qref.2018.11.009

[42] Samadi A Rad EH. Determinants of healthcare expenditure in economic cooperation organization (ECO) countries: evidence from panel cointegration tests. Int J Health Policy Manag. (2013) 1:63–8. doi: 10.15171/ijhpm.2013.10, PMID: 24596838 PMC3937933

[43] Folahan D Awe A. An assessment of health expenditure determinants in Nigeria. IOSR J Econ Finance. (2014) 3:23–30. doi: 10.9790/5933-03212330

[44] Khan HN Razali RB. Modeling determinants of health expenditures in Malaysia: evidence from time series analysis. Front Pharmacol. (2016) 7:1–7. doi: 10.3389/fphar.2016.00069 27065860 PMC4811951

[45] Boachie MK Mensah IO Sobiesuo P Immurana M Iddrisu AA Kyei-Brobbey I. Determinants of public health expenditure in Ghana: a cointegration analysis. J Behav Econ, Finance, Entrepreneurship, Account Transport. (2014) 2:35–40. doi: 10.12691/jbe-2-2-1

[46] Bayar Y Gavriletea MD Pintea MO Sechel IC. Impact of environment, life expectancy and real GDP per capita on health expenditures: evidence from the EU member states. Int J Environ Res Public Health. (2021) 18:13176. doi: 10.3390/ijerph182413176, PMID: 34948785 PMC8702070

[47] Herwartz H Theilen B. The determinants of health-care expenditure: new results from semiparametric estimation. Health Econ. (2010) 19:964–78. doi: 10.1002/hec.1540, PMID: 19662662

[48] Herwartz H Theilen B. The determinants of health care expenditure: testing pooling restrictions in small samples. Health Econ. (2003) 12:113–24. doi: 10.1002/hec.700, PMID: 12563659

[49] Hitiris T Posnett J. The determinants and effects of health expenditure in developed countries. J Health Econ. (1992) 11:173–81. 10122977 10.1016/0167-6296(92)90033-w

[50] Olasehinde N Olaniyan O. Determinants of household health expenditure in Nigeria. Int J Soc Econ. (2017) 44:1694–709. doi: 10.1108/IJSE-12-2015-0324

[51] Magazzino C Mele M. The determinants of health expenditure in Italian regions. Int J Econ Financ. (2012) 4:61–72. doi: 10.5539/ijef.v4n3p61

[52] Yetim B İlgün G Çilhoroz Y Demirci Ş Konca M. The socioeconomic determinants of health expenditure in OECD: an examination on panel data. Int J Healthc Manag. (2021) 14:1265–9. doi: 10.1080/20479700.2020.1756112

[53] Hosoya K. Determinants of health expenditures: stylized facts and a new signal. Mod Econ. (2014) 5:1171–80. doi: 10.4236/me.2014.513109

[54] Akca N Sonmez S Yilmaz A. Determinants of health expenditure in OECD countries: a decision tree model. Pakistan J Med Sci. (2017) 33:1490–4. doi: 10.12669/pjms.336.13300, PMID: 29492084 PMC5768850

[55] Okunade AA. Analysis and implications of the determinants of healthcare expenditure in African countries. Health Care Manag Sci. (2005) 8:267–76. doi: 10.1007/s10729-005-4137-5, PMID: 16379410

[56] Nghiem SH Connelly LB. Convergence and determinants of health expenditures in OECD countries. Heal Econ Rev. (2017) 7:1–11. doi: 10.1186/s13561-017-0164-4 PMC5560333 28819772

[57] Martín JJM Lopez P del Amo GM Garcia DC. Review of the literature on the determinants of healthcare expenditure. Appl Econ. (2011) 43:19–46. doi: 10.1080/00036841003689754

[58] Prieto DC Lago-Peñas S. Decomposing the determinants of health care expenditure: the case of Spain. Eur J Health Econ. (2012) 13:19–27. doi: 10.1007/s10198-010-0276-9, PMID: 20853126

[59] Grossman M. The demand for health; a theoretical and Emprical investigation. New York: National Bureau of Economic Research (1972).

[60] Matteo DL. The macro determinants of health expenditure in the United States and Canada: assessing the impact of income, age distribution and time. Health Policy. (2015) 71:23–42. doi: 10.1016/j.healthpol.2004.05.007, PMID: 15563991

[61] TÜİK. (2023). Türkiye İstatistik Kurumu. Available online at: http://www.tuik.gov.tr/MicroVeri/HBA_2019/english/index.html (Accessed March 03, 2024).

[62] Wooldridge JM. Econometric analysis of cross section and panel data. 2nd ed. England: MIT Press (2010).

[63] Çebi Karaaslan K Oktay E Alkan Ö. Determinants of household saving behaviour in Türkiye. Sosyoekonomi. (2022) 30:71–90. doi: 10.17233/sosyoekonomi.2022.01.04

[64] Tatar M Ozgen H Sahin B Belli P Berman P. Informal payments in the health sector: a case study from Turkey. Health Aff. (2007) 26:1029–39., PMID: 17630446 10.1377/hlthaff.26.4.1029

[65] Sosyal Güvenlik Kurumu. (2025). Gençler Genel Sağlık Sigortasından Nasıl Yararlanmaktadır? Available online at: https://www.sgk.gov.tr/Content/Post/46f39449-681c-4979-9780-9432d56c1478/Gencler-Genel-Saglik-Sigortasindan-nasil-yararlanmaktadir-2023-01-25-02-09-19 (Accessed April 04, 2025).

[66] Sosyal Güvenlik Kurumu. (2025). yılı için genel sağlık sigortası prim tutarı ne kadardır? Available online at: https://www.sgk.gov.tr/Content/Post/146ba5fa-f757-4fc1-b316-30859c42d21e/2025-yili-icin-genel-saglik-sigortasi-prim-tutari-ne-kadardir-2025-02-06-09-59-38 (Accessed April 04, 2025).

[67] T.C. Sağlık Bakanlığı Sağlığın Geliştirilmesi Genel Müdürlüğü. Türkiye Sağlık Okuryazarlığı Düzeyi ve İlişkili Faktörleri Araştırması. Ankara: Özyurt Matbaacılık (2018).

[68] T.C. Sağlık Bakanlığı Sağlığın Geliştirilmesi Genel Müdürlüğü. Türkiye Sağlık Okuryazarlığı Düzeyi ve İlişkili Faktörleri Araştırması. Ankara: Semih Ofset (2024).

